# Phytoplankton community structuring and succession in a competition-neutral resource landscape

**DOI:** 10.1038/s43705-021-00011-5

**Published:** 2021-04-14

**Authors:** Michael J. Behrenfeld, Emmanuel S. Boss, Kimberly H. Halsey

**Affiliations:** 1 Department of Botany and Plant Pathology, Oregon State University, Corvallis, OR USA; 2 School of Marine Sciences, University of Maine, Orono, ME USA; 3 Department of Microbiology, Oregon State University, Corvallis, OR USA

**Keywords:** Microbial ecology, Microbial ecology

## Abstract

Phytoplankton community composition and succession affect aquatic food webs and biogeochemistry. Resource competition is commonly viewed as an important governing factor for community structuring and this perception is imbedded in modern ecosystem models. Quantitative consideration of the physical spacing between phytoplankton cells, however, suggests that direct competition for growth-limiting resources is uncommon. Here we describe how phytoplankton size distributions and temporal successions are compatible with a competition-neutral resource landscape. Consideration of phytoplankton-herbivore interactions with proportional feeding size ranges yields small-cell dominated size distributions consistent with observations for stable aquatic environments, whereas predator–prey temporal lags and blooming physiologies shift this distribution to larger mean cell sizes in temporally dynamic environments. We propose a conceptual mandala for understanding phytoplankton community composition where species successional series are initiated by environmental disturbance, guided by the magnitude of these disturbances and nutrient stoichiometry, and terminated with the return toward a ‘stable solution’. Our conceptual mandala provides a framework for interpreting and modeling the environmental structuring of natural phytoplankton populations.

## Introduction

A central focus in ecology is understanding community composition and succession, as these attributes influence food web structure and trophic energy transfer.^1–3^ In aquatic systems, characteristics of the phytoplankton community also have strong biogeochemical consequences. In particular, stable and low-nutrient regions tend to be dominated by smaller phytoplankton species that support complex food webs with efficient elemental recycling in the upper sunlit photic layer. In contrast, the biomass of larger phytoplankton is often enhanced in more dynamic environments, giving rise to shorter food chains and increasing material export to depth.^4–8^ Understanding the basic mechanisms governing size structuring of phytoplankton communities provides insight on potential impacts of environmental change and implications for biogeochemistry and higher trophic level production (e.g., fisheries).

An assumption that direct competition for growth-limiting resources is an important force in ecosystem structuring has guided interpretations of phytoplankton communities toward a focus on competitive phenologies (e.g., size-dependent nutrient uptake kinetics, contrasting photoadaptation strategies, etc.)^9–11^ and this interpretation is imprinted in modern ecosystem models.^12,13^ However, in nearly all natural aquatic environments, phytoplankton populations are constrained to such an extent by tight food web coupling and resource supply (light, nutrients) that individual cells are, on average, sparsely distributed (as a more tangible analogy, if the average body length spacing between phytoplankton is scaled up to the size of root systems for typical 12 m tall deciduous trees, the average spacing between neighboring trees would be >1 km [Supplementary Information]). When the nutrient depletion zones around phytoplankton are considered, the physical spacing between individuals indicates that neighboring cells rarely compete directly with one another for growth-limiting resources.^14,15^ In other words, at the scale of phytoplankton, these resources are experienced as diffuse fields while phytoplankton themselves are discrete, distantly spaced entities. This condition of non-overlapping nutrient uptake fields between individuals is referred to herein as the ‘competition-neutral resource landscape’ and it suggests that factors other than resource acquisition differences may play dominant roles in structuring phytoplankton communities. Here, we examine characteristics of phytoplankton community structure and succession largely from the perspective of predator–prey relationships functioning in this competition-neutral resource landscape. We begin with an ecological explanation for the conserved phytoplankton size distribution observed in temporally stable aquatic environments and then describe processes that ‘disturb’ populations away from this stable distribution. These concepts are then knit together into a conceptual mandala depicting phytoplankton successional sequences that can inform interpretations of regionally contrasting contemporary phytoplankton populations and predictions of future change.

## Size structuring of stable phytoplankton communities

**Fig. 1:**
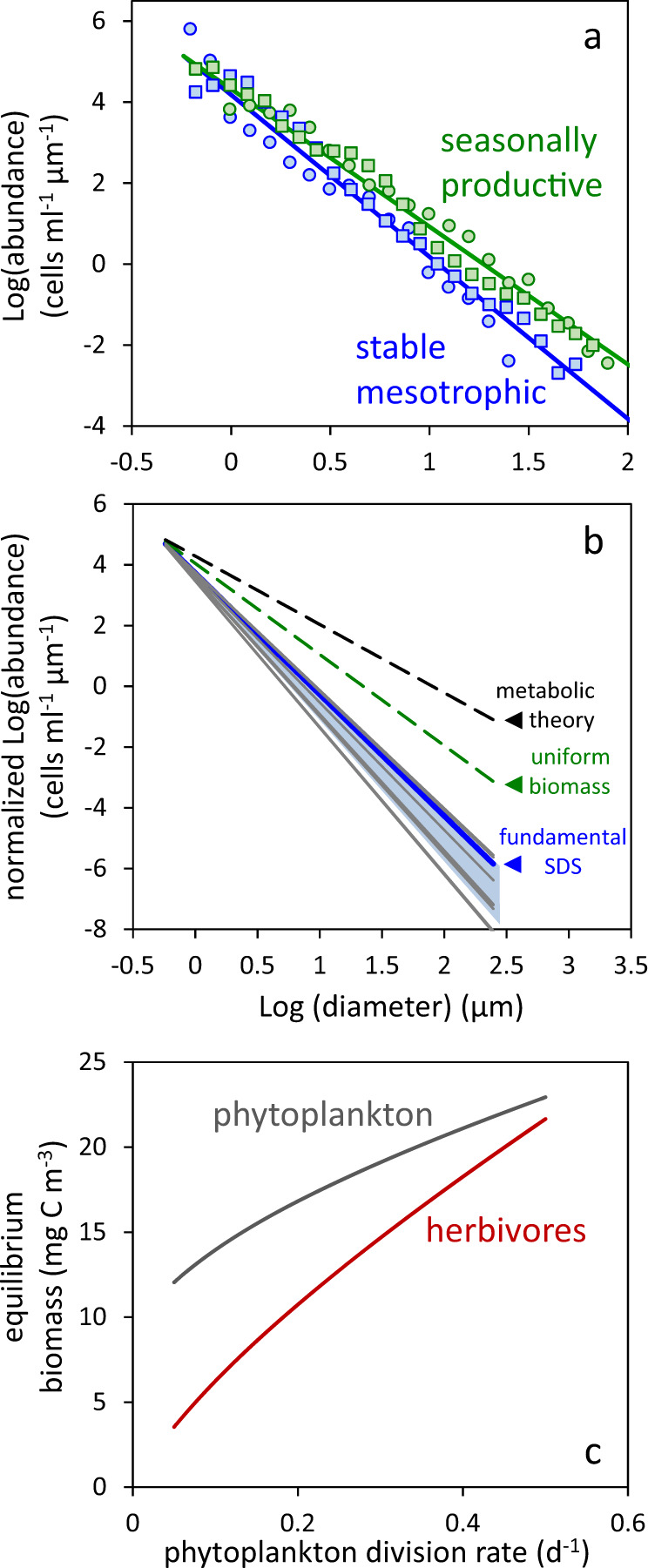
Phytoplankton size distributions and modeled equilibrium solutions for plankton biomass. **a** Examples of measured phytoplankton size distributions for (blue symbols) stable mesotrophic conditions (chlorophyll concentration ~ 0.1 mg m^−3^) and (green symbols) seasonally productive regions (chlorophyll concentration ~ 1 mg m^−3^). (blue line) SDS = −4. (green line) SDS = −3.4. Data from (circles) Marañón^24^ and (squares) unpublished data from the NAAMES program.^97^ **b** Phytoplankton size distributions (dashed black line) predicted from metabolic theory (SDS = −2.25), (dashed green line) for uniform biomass in all size classes (SDS = −3), and (heavy blue line) predicted for a competition-neutral resource environment with proportional grazing size ranges (SDS = −4). Light blue shading indicates SDS range for stable environments when phytoplankton division rates (*µ*) and herbivore ingestion efficiency (*c*_2_) and predatory loss (*c*_3_) rates are assumed to be size dependent [Supplementary Information] (gray lines). Examples of measured phytoplankton size distributions in stable, nutrient-limited regions [from ^23^]. All size distributions are normalized to 10^4^ cells ml^−1^ at the 0.5 µm size bin. **c** Modeled (Eqs. 1 and 2) equilibrium phytoplankton and herbivore biomass as a function of phytoplankton division rate [from ^29^].

Energy transfer efficiencies between predators and prey critically influence body size versus abundance relationships across trophic levels.^16–18^ Metabolic theory has provided a mechanistic interpretation of such relationships,^3^ where observed quarter-power allometric scaling is attributed to fractal resource uptake and distribution systems within organisms.^19,20^ However, phytoplankton lack such distribution systems and alternative mechanisms for community structuring within their trophic level need to be considered. A field-based observation of primary importance with respect to phytoplankton communities is that their size distribution generally exhibits a slope of approximately −4 between the logarithm of cell number concentration per unit length and the logarithm of cell diameter (e.g., blue symbols and lines in Fig. 1a, b).^21,22^ Hereafter, we will refer to this distribution as the ‘fundamental’ phytoplankton size distribution slope (SDS). In temporally stable aquatic environments (e.g., nutrient-impoverished tropical and subtropical oceans), variability around this fundamental SDS is remarkably constrained between roughly −3.9 and −4.8 (gray lines in Fig. 1b).^23,24^ These observations are inconsistent with metabolic theory, which predicts a phytoplankton SDS of −2.25^3^ (black dashed line in Fig. 1b). An alternative explanation for the fundamental phytoplankton SDS can be investigated using established phytoplankton-herbivore-predator relationships found in ecosystem models.

While a diversity of model formulations exists describing plankton dynamics, a simple set of equations is sufficient here to illustrate the temporal balance between the biomass of phytoplankton in a given size class (*P*_*i*_) and their herbivorous predators (*H*):

1 $$\frac{{dP_i}}{{dt}} = \mu P_i - c_{1,i}P_iH$$

2 $$\frac{{dH}}{{dt}} = c_{1,i}c_2P_iH - c_3H^2$$

where *µ* is phytoplankton division rate and parameters *c*_1_–*c*_3_ are herbivore grazing rate, ingestion efficiency, and predatory loss rate, respectively.^25–27^ While our model equations do not take into account numerous attributes of plankton ecosystems such as grazing saturation, grazing thresholds, and other non-linear behaviors, they do yield the important results that values for *P*_*i*_ and *H* are proportional to *µ* (Fig. 1c)^28,29^ and that they are size-independent for *P*_*i*_ when *µ* and the *c* parameters are held constant across size classes [Supplementary Information]. In other words, the model predicts phytoplankton biomass to vary with division rate and, for a given division rate, to be equivalent in each size bin. If these size bins have the same absolute range in cell diameters, then this result implies a logarithmically transformed phytoplankton SDS of −3 (dashed green line in Fig. 1b) because biomass per cell scales approximately with cell volume [Supplementary Information]. While this SDS is steeper than that predicted by metabolic theory, it is still significantly shallower than that observed for natural phytoplankton communities in temporally stable environments (blue and gray lines in Fig. 1a, b).

While phytoplankton predators exhibit a diversity of body sizes, with some even comparable to their prey,^30–32^ the absolute phytoplankton size range grazed upon by these herbivores is generally proportional to their average prey size.^32–37^ In other words, grazers of very small phytoplankton typically have a narrower absolute prey size range than grazers of large phytoplankton. This phenomenon is accounted for in Eqs. (1) and (2) by making the span of phytoplankton sizes in successive *i* bins proportional to size [Supplementary Information] and this adjustment alone tips the logarithmically transformed phytoplankton SDS to the observed −4. In other words, the broader prey size range of grazers feeding on large phytoplankton yields a steady-state prey biomass of lower average concentration at each size within that range than that for grazers of smaller phytoplankton. It is worth noting here that some grazers feed wholesale across the phytoplankton size domain (e.g., gelatinous tunicates feeding with mucous webs,^38,39^) rather than in proportion to average prey size. Feeding by these organisms will positively tilt the phytoplankton SDS to a value between −4 and −3 in a manner proportional to their relative contribution to total phytoplankton loss rates.

The important conclusion from above is that the fundamental SDS under stable growth conditions can be accounted for without invoking direct size-dependent competition for growth-limiting resources between individual phytoplankton. However, a competition-neutral landscape does not imply that biological rates are necessarily uniform across sizes. If we consider a nutrient-limited system where phytoplankton of all sizes are capable of fully depleting their limiting resource at the rate it is made available around the cell’s zone of influence, then the absolute nutrient flux acquired by a given cell will be proportional to its surface area which, when divided by cell quota for that nutrient, implies a slower division rate for larger cells. Under such conditions, faster division in smaller phytoplankton is expected to cause an increase their relative abundance (Fig. 1c).^28,29^ The significance of this phenomenon can be evaluated with our model by imposing a strong size-dependent function for *µ* [Supplementary Information], but this modification only yields a minor tipping of the SDS from −4 to −4.1. This insensitivity of the SDS to size-dependent differences in *µ* results because increased division rates are accompanied by nearly equivalent increases in loss rates.^28,29^ Alternatively, we can implement significant size-dependent parameterizations for herbivore ingestion efficiency (*c*_2_) and predatory loss rate (*c*_3_) in the model [Supplementary Information], but again these modifications only modestly broaden the SDS range. When all three size-dependent parameterizations are implemented (*µ, c*_2_, *c*_3_), the predicted phytoplankton SDS decreases to just −4.6, which is well within the range of variability observed in stable aquatic ecosystems (compare blue shading and grey lines in Fig. 1b).

**Fig. 2:**
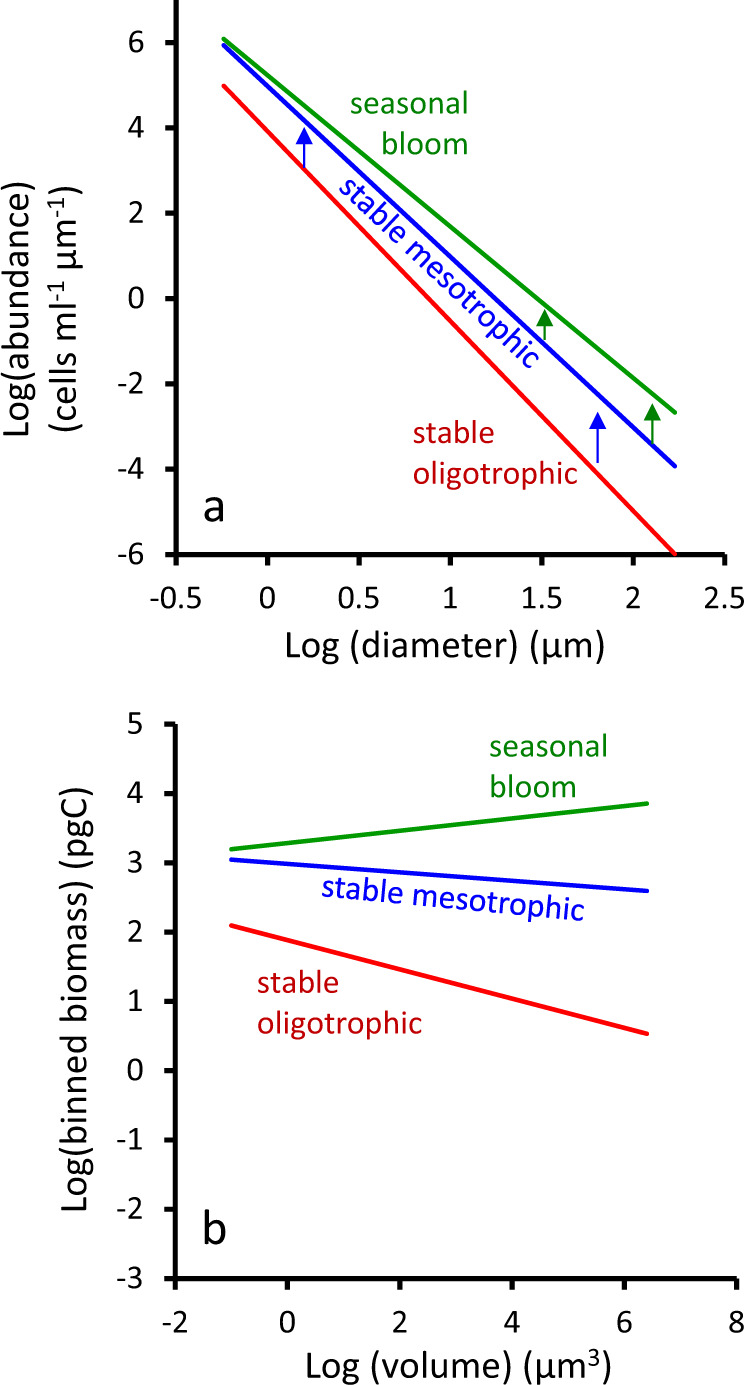
Fundamental behavior of phytoplankton size distributions and biomass in contrasting environments. **a** Canonical phytoplankton size distribution for (red line) a stable oligotrophic systems where abundance of all size classes is suppressed by low nutrients, (blue line) a stable mesotrophic system where nutrients are elevated, and (green line) a phytoplankton bloom where a seasonally variable environment creates favorable changes in growth conditions that preferentially enhance larger phytoplankton due to impacts on predator–prey relationships. **b** Same data as in (**a**) but with (*x* axis) cell diameter converted to cell volume and (*y* axis) cell abundance converted to biomass assuming a constant biomass per unit volume^98^ and integrated over logarithmically spaced bins.^21^

The central conclusion from the forgoing considerations is that the size structure of phytoplankton communities in temporally stable environments deviates from that of a simple uniform nutrient allocation across sizes (i.e., SDS of −3) predominantly because of size-dependent attributes of predator–prey relations (most notably, proportional feeding ranges), whereas size-dependent phenologies linked to nutrient acquisition and growth rate play a secondary role (for the current simulations, a shift in SDS of −0.1). Based on these findings, our expectation is that phytoplankton size distributions will be essentially independent of limiting resource supply under steady-state conditions because nutrient supply rate does not directly impact the predator–prey interactions predominantly governing community structuring. This conclusion is consistent with observations from stable oligotrophic to mesotrophic^40^ systems where the relative abundance of pico-, nano-, and micro-phytoplankton remains relatively unchanged across more than an order of magnitude variation in chlorophyll concentration from 0.01 to ~0.3 mg m^−3^ [e.g., ^23,41,42^]. In other words, between-region differences in nutrient supply in temporally stable aquatic environments are predominantly reflected by a general upregulation of the entire plankton community (i.e., a relatively constant SDS; shift from the red to blue line in Fig. 2a), where nutrients sequestered in biomass are a significant fraction of total nutrient load, rapid biomass recycling sustains phytoplankton growth rates, and dissolved nutrient concentrations are relatively invariant between regions. Revising the idea of Barber and Hiscock,^6^ we might metaphorically describe this size-independent response to nutrient supply in stable aquatic environments as “A higher tide lifts all phytoplankton“.

## Phytoplankton size structuring in dynamic environments

When growth conditions in an aquatic system vary significantly in time, phytoplankton size distributions often deviate from the fundamental SDS discussed above. Specifically, a relative increase in larger-sized species causes the SDS to become less steep (e.g., green symbols and line in Fig. 1a).^24,41–43^ This shift results from a size-dependent disequilibrium between phytoplankton division (*µ*) and loss (*l*) rates in nature. In aquatic ecosystems, short (order, days) temporal lags exist between changes in phytoplankton division rates and grazing by herbivores. These temporal lags become expressed in phytoplankton biomass when resource supplies are perturbed on times scales spanning from storm fronts to seasons.

A simplification of Eq. 1 expresses the rate of change in phytoplankton biomass (*r*) as:

3 $$\frac{1}{P}\frac{{dP}}{{dt}} = r_t = \mu _t - l_t$$

where the *t* subscript refers to time. In the tightly coupled but time-lagged plankton predator–prey system, the value of *l*_*t*_ can be equated to a prior value of *µ,*^28,29,44^ yielding the revised expression:

4 $$r_t = \mu _t - \mu _{t - j} = \frac{{d\mu }}{{dt}}\,\Delta t$$

where *j* is the time-lag between division and loss rates. Equation 4 implies that phytoplankton biomass increases when division rates accelerate and decreases when they decelerate.^28,29,44^ As described below, this dependency ties phytoplankton community composition, and thus the size distribution, in a variable growth environment to size-dependent differences in predator–prey time lags (*j*).

For the phytoplankton, predator–prey coupling is tighter for smaller phytoplankton (i.e., the value of *j* increases with size) because predators of large phytoplankton often have complicated life cycles that delay responses to prey abundance and because handling times generally increase with prey size.^45–48^ This difference gives larger phytoplankton an advantage when temporal changes in growth conditions are sufficiently prolonged that differences in predator–prey time lags (*j*) can become expressed at the community level.^49^ In addition, strongly seasonal aquatic systems are typically associated with periods of elevated nutrients. Bloom-forming species with an ability to greatly accelerate their division rates then have an additional advantage.^49^ Diatoms, with their many larger-sized representatives, include a diversity of species with a particular proclivity for rapid and prolonged increases in division rate.^49^ Thus, temporal variations in growth conditions shift the size structure of phytoplankton communities (positive tilting of the SDS from blue lines to green lines in Figs. 1a and 2a) because size-dependent differences in predator–prey relations and evolved ‘bloom-forming’ physiologies slightly favor larger species.

## Mandalas

**Fig. 3:**
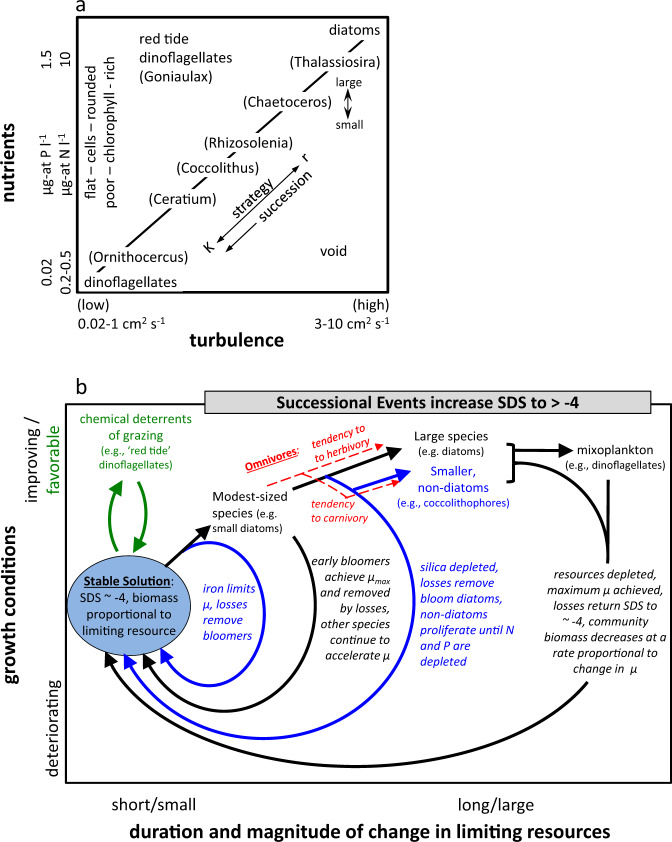
Phytoplankton community composition and succession mandalas. **a** Margalef’s Mandala redrawn from the original publication^51^ where the two determinant axes are turbulence and nutrient concentration [either nitrogen (N) or phosphorous (P)]. With respect to the successional sequence proposed, Margalef also noted characteristics of phytoplankton cell shape, community chlorophyll concentration, and *K* versus *r* life strategy.^1^ **b** Proposed mandala where the determinant axes are the duration and magnitude of change in limiting resources and the trajectory of growth conditions. (blue shaded area) ‘Stable Solution’ where total phytoplankton biomass varies with nutrient load but the SDS is constrained to approximately −4. (blue and black arrows) Successional sequence under variable growth conditions resulting from accelerations and decelerations in phytoplankton division rate that impact the balance between division and loss rates and cause a positive tipping of the SDS. (rightward pointing black arrows) Succession of bloom-forming species where large cells ultimately dominate bloom biomass if the amplitude of change in growth conditions is sufficiently large to allow the full successional series, which may then be followed by a rise in mixotrophic species. (leftward pointing blue and black arrows) Variations on the return pathway to the ‘Stable Solution’ associated with different nutrient stoichiometries and cell sizes. The rightmost blue path is associated with Si limitation of diatoms and a shift in feeding tendencies of omnivores (red dashed arrows). (green arrows) An alternative succession scenario where blooming in a favorable high-nutrient but stable environment favors species that chemically suppress grazing (e.g., toxic algal blooms of ‘red tide’ dinoflagellates).

The term ‘mandala’ originally referred to a geometric pattern representing spiritual or cosmic structure, but in science it is used today in reference to a diagram or chart capturing the essence of a particular phenomenon or process.^50^ Ramón Margalef^51^ posited a simple and elegant explanation for the seasonal succession of phytoplankton species that has served as a cornerstone in aquatic ecology and is known as ‘Margalef’s mandala’. The determinate axes of the mandala are strength of ‘turbulence’ and ‘nutrient concentration’, where diatoms with a propensity to sink require high turbulence to remain in suspension and large diatoms require the highest nutrients to flourish (Fig. 3a). As nutrients and turbulence diminish, the mandala depicts phytoplankton communities as transitioning to dominance by coccolithophores and finally to swimming dinoflagellates (Fig. 3a). Outside of the primary successional line, Margalef identified low-turbulence, high-nutrient conditions as favoring blooms of red tide dinoflagellates and he considered high-turbulence, low-nutrient conditions as nonexistent (i.e., ‘void’, Fig. 3a) (this quadrant was later suggested to represent iron-limited regions.^52^) Since its publication, Margalef’s mandala has seen both simplifications and embellishments,^52–55^ with one recent revision increasing the determinant axes from 2 to 12 dimensions.^50^

While a mandala is not expected to capture the full details of its subject, our understanding of plankton ecosystems has matured sufficiently since Margalef’s seminal work^53^ that it may be time to retire this framework for an alternative schematic. We now know, for example, that turbulence is not required to sustain diatoms in the sunlit surface layer, as even the largest diatoms maintain neutral buoyancy when healthy.^56–60^ We also know that small, non-diatom species typically dominate high latitude phytoplankton communities during winter when turbulence is maximal and nutrient loads are high.^61,62^ Diatom dominance instead typically occurs later when turbulence is greatly diminished. The succession among diatom species during blooms is also commonly from smaller species when nutrient levels are high to larger species as nutrients become depleted.^63^ In addition, technological developments since Margalef’s time have revealed the numerical dominance of very small phytoplankton under all conditions and that species diversity (including diatoms) is comparable in low-nutrient oligotrophic waters and high latitude bloom-forming regions.^53,64^

We propose an alternative mandala for phytoplankton community structure and succession based on the size distribution, ‘higher tide’, and eco-physiological principles described above (Fig. 3b). In our mandala, the determinant axes are now the ‘duration and magnitude of change in limiting resources’ and the direction in which ‘growth conditions’ are changing (i.e., improving or deteriorating). We envision the default attractor for all phytoplankton communities to be the fundamental SDS of −4 discussed above (blue shaded circle in Fig. 3b). Primarily the biomass, not the SDS, of this ‘Stable Solution’ is expected to vary between regions in proportion to the supply rate of limiting resource (i.e., the ‘higher tide’ concept), so long as the physical environment is relatively stable in time (e.g., oligotrophic to mesotrophic tropical and subtropical open ocean regions).

In our schematic, departures from the ‘Stable Solution’ (rightward pointing blue and black arrows in Fig. 3b) are driven by temporal variations in resource supply, as may be associated with changes in turbulence, riverine nutrient supply, solar irradiance, coastal upwelling, or other forcings. These departures result in a successional sequence that favors larger phytoplankton species and thus positively tilts the SDS to >−4. In other words, changes are occurring within all phytoplankton size classes (Figs. 1a and 2a) over a successional sequence, but our mandala focuses on those changes that impact the community size distribution. To the right of the ‘Stable Solution’ (Fig. 3b), improving growth conditions cause accelerations in phytoplankton division rate that are temporally lagged by loss rates and thus result in increased phytoplankton concentrations. If the magnitude of resource variability is modest, the likely outcome is a proliferation of modest-sized species with both an enhanced ability to accelerate division rates and a high maximum division rate, *µ*_max_ (e.g., small bloom-forming diatoms).^49^ The value of *µ*_max_ is important here because it defines when the period of increasing division rate must end for a given species under improving growth conditions. Size-dependent differences in division rates prior to improving growth conditions (*µ*_min_) are also critical because the difference between *µ*_min_ and *µ*_max_ defines the duration of accelerations in division rate and, thus, biomass accumulation for a given species. Rapid accelerations in the division rate of modest-sized species, however, also means that *µ*_max_ might be achieved in these ‘early bloomers’ before resources are depleted. In such a case, which becomes more likely as the magnitude of resource variability increases, loss rates catch up with division rates of the ‘early bloomers’ and terminate their bloom, while larger and more slowly accelerating species with greater predator–prey lags continue to have an opportunity to proliferate (e.g., large diatoms).^53^ A reduction in inorganic nutrients or light brings an end to the late-blooming obligate photoautotrophs, but an opportunity may still exist for the continued accumulation of mixoplankton (e.g., dinoflagellates, some haptophytes, silicoflagellates) that can both photosynthesize and tap into particulate organic matter amassed over the earlier blooming phases (Fig. 3b).^65,66^

Within our conceptual framework, a variety of paths return phytoplankton communities toward the ‘Stable Solution’ (leftward pointing blue and black arrows in Fig. 3b). The different paths reflect the magnitude of limiting resource variability (*x* axis) and are taxonomically dependent. For example, the magnitude of resource variability at higher latitudes is bounded by the difference between the winter light-limited *µ*_min_ and either the nutrient- or light-limited *µ*_max_ allowed in the subsequent spring or summer. In the eastern subarctic Atlantic where deep winter mixing heavily enriches the surface layer with micro- and macro-nutrients, this magnitude may be sufficiently large to support the full succession from small-to-large diatom blooms. In contrast, the Southern Ocean, with comparable or even deeper winter mixing, becomes iron-deficient by early spring,^67,68^ leading to a lower *µ*_max_ and a truncated succession (leftmost blue return arrow in Fig. 3b). Shallower winter mixing in the western subarctic Atlantic similarly has the potential to reduce the *µ*_min_-to-*µ*_max_ difference (due to enhanced winter division rates), resulting in a bloom climax dominated by smaller species (i.e., the full procession to large diatoms is precluded).^49,62^

Nutrient stoichiometry also plays an important role in phytoplankton succession. For example, a nutrient supply that is deficient in silica will ‘short-circuit’ a small-to-large diatom succession (rightmost blue return arrow in Fig. 3b). Residual N and P not taken up by the diatoms thus create a ‘window of opportunity’ for other non-diatoms species to proliferate, such as moderate-sized coccolithophorids (rightward pointing blue arrow in Fig. 3b). Normally, heavy grazing pressure constrains the abundance of these species, but failure of a large diatom bloom to materialize can drive omnivorous grazers to switch their mode of feeding from diatom herbivory to predation on the micrograzers who feed upon smaller phytoplankton, effectively enabling an accumulation in moderate-sized phytoplankton through a trophic cascade effect (dashed red arrows in Fig. 3b).

The successional sequence thus described focuses on environmentally driven imbalances in predator–prey relations governed by temporal changes in phytoplankton division rates. An alternative to ‘outrunning’ predators for some phytoplankton species has been to develop chemical deterrents that effectively shift grazing toward selection of more palatable species.^69–74^ This approach may be particularly important for dinoflagellates during the later stages of our primary successional sequence or in areas of shallow mixing and elevated nutrients (green arrows in Fig. 3b).^75^

## Discussion

A stark contrast exists in the ecological and biogeochemical functioning of oligotrophic and productive aquatic systems because their modest differences in phytoplankton size distributions are greatly amplified by the relationship between cell size and volume. The prominence of smaller cells, both in abundance (Figs. 1a, b and 2a) and biomass (Fig. 2b), in oligotrophic systems is associated with increased food web complexity, enhanced nutrient recycling, and less efficient carbon export to depth.^7,8^ In contrast, while more productive systems are still numerically dominated by smaller phytoplankton (Figs. 1a and 2a), the balance of biomass lies in the larger fraction (Fig. 2b). Consequently, shorter trophic pyramids can be supported in such systems, yielding higher energy transfer efficiencies and enhanced material export to depth.^4–8^

If we conceive of phytoplankton as a diffusive field defined by an elemental stock (e.g., N or C) where resources are a commodity uniformly accessible across size classes, as is common in modern ecosystem models, then detailed nutrient uptake traits relevant to resource competition play an enhanced role in structuring community composition.^9,11,76,77^ Maintaining species diversity in such a construct benefits from temporal variations in growth conditions that ensure competitive exclusion is not too severe.^78^ Considerable experimental and theoretical work has been conducted to evaluate competition among phytoplankton [^79–84^, but see.^15^] If instead we adopt a view of phytoplankton communities where natural cell densities sufficiently distance individuals that resources are rarely a shared commodity at a given moment in time,^14,15^ then the problem of community structure and succession refocuses on trophic interactions in tightly coupled food webs. Here, we suggest that attributes of predator–prey relationships functioning in a competition-neutral resource landscape can account for spatial and temporal properties of natural phytoplankton communities. However, our analysis of community structure is constrained to the broad property of phytoplankton size distributions and does not address species diversity within a given size range. It may be that addressing this latter issue will reveal that advantages gained in resource acquisition from unique physiological traits play a greater role.

The competition-neutral resource environment envisioned here reflects the overall nature of the phytoplankton landscape and does not imply a uniform spatial distribution of cells nor an absence of any interactions between individuals. Certainly, processes of cell division, predation, cell sinking or rising, swimming, and turbulence all cause cells to continuously move relative to each other, resulting in nutrient depletion zones that temporarily overlap between individuals and, occasionally, causing cells to ‘bump into each other’, stick, and form aggregates.^85–89^ The critical attribute of the competition-neutral landscape is that nutrient uptake by one cell at a given moment in time generally does not directly impact the nutrient environment experienced by its relatively distant neighbors. Because the turnover rate of phytoplankton biomass is fast (order, days), any signatures of different nutrient uptake affinities in the local environment around individuals are erased through recycling, turbulence, and diffusion. If this view is valid, then it raises a subsequent question of why differences in nutrient uptake efficiencies have evolved between species? Perhaps the answer to this question is also tied to predator–prey relationships. Specifically, while phytoplankton may not directly compete with one another for resources, species that can extract more of a limiting resource from their immediate environment can sustain higher growth rates than those that cannot. If the loss rate within a given size class is species-independent, then species with higher division rates may secure a greater fraction of biomass within their size class. An alternative to the rapid-division strategy that has an equivalent outcome is for a species to specifically diminish its own loss rates, with the example given above being the production of chemicals that deter grazers. Thus, while physiological inventions impacting the predator–prey balance for a given species may have neither a significant impact on the overall size distribution of a phytoplankton community nor the immediate growth environment of neighboring cells, they may play an important role in a species’ resilience to their own population’s decline or local extinction. It may further be envisioned how such physiological advantages, when played out over sufficient time, could effectively partition aquatic environments into spatially distinct communities.

Recognizing the significance of spatial distancing on cell-to-cell resource competition^14,15^ focused our analysis on the role of phytoplankton-herbivore relations with regard to community size distribution and succession. These relations are but one element in a multitude of biotic interactions that appear to dominate the more detailed structuring of plankton communities.^90^ In some cases, the physiological underpinning of such interactions and their significance to community assembly are unclear. For example, many phytoplankton species lack the ability to produce certain vitamins and instead rely on a supply from other members of the community.^91,92^ Does this absence of central biosynthetic pathways represent a significant selective advantage or is it simply ‘permissible’ so long as an external source is available? Another example of downselected physiological competence is provided by *Prochlorococcus*, which has lost the ability to utilize nitrate and tolerate low temperature. Here, the advantage of genome streamlining is clearer as it has minimized cell size beyond that of all other phytoplankton and opened a new niche in the size distribution that allows numerical dominance in tropical and subtropical oceans.^93^ Other adaptations have also evolved that effectively violate conditions of the competition-neutral resource environment. For example, there is a growing recognition that diverse symbiotic relationships exist between unicellular plankton. These relationships allow physical limits on resource acquisition to be alleviated, be it for specific compounds in a phytoplankton-bacteria association^94^ or general organic supplement and nutrient exchange in a phytoplankton-herbivore association.^95,96^ In other words, symbiosis may provide a competitive advantage by discretizing an otherwise diffuse resource.

Based on our analyses, we propose a mandala depicting phytoplankton community structure and succession (Fig. 3b). While we have noted examples of phytoplankton types that may be associated with different stages of the successional sequence, the mandala focuses more on underlying predator–prey relations and cell physiologies influencing these relationships. Importantly, these key physiological attributes may not be unique to specific taxa, but rather may be shared across many species or even broad taxonomic groups. Conceptual frameworks such as our mandala and that of Margalef can guide understanding of global ecosystems and can function as a basis for model development and prediction. Expectations of how environmental change will impact aquatic ecosystems depend on our conception of such system functions in the contemporary world. If, for example, we view direct competition between individuals as important to community structuring, we might expect a reduction in surface nutrients under a warming climate to selectively favor smaller species due to their advantageous surface-to-volume ratios. In contrast, the competition-neutral resource landscape envisioned herein suggests that this same warming scenario will decrease total phytoplankton biomass but with only a minor impact on the size distribution. These contrasting expectations have profoundly different implications for aquatic food webs and biogeochemistry, emphasizing the crucial nature of understanding growth and loss processes at the discrete level of phytoplankton cells.

## Supplementary Information

Supplementary Information
