# Supplementary material for: Phytoplankton community structuring and succession in a competition-neutral resource landscape
Source: ISME Commun. 2021 Apr 14;1:12. doi: 10.1038/s43705-021-00011-5 (PMC9645248; doi:10.1038/s43705-021-00011-5)
Supplement: Supplementary file 1 — Supplementary Information [file 43705_2021_11_MOESM1_ESM.pdf]

Supplementary Information

This file includes supplemental text regarding calculations of our tree-spacing analogy and regarding the model equations used to generate results shown in figure 1b and 1c of the manuscript, “Phytoplankton community structuring and succession in a competition-neutral resource landscape ”, by Behrenfeld *et al*.

Tree-spacing Analogy

The spatial scales relevant to phytoplankton interactions can be difficult to comprehend because they are so vastly different that those that humans function in. In the beginning of our manuscript, we therefore provided an analogy for the average spatial distancing between neighboring phytoplankton in terms of equivalent relative distances between the roots of neighboring trees. For this analogy, we conservatively assumed an extremely high phytoplankton concentration of 10^{13} cells m^{-3}. At this concentration, the average distance between the centers of individual cells is ~45 μm. The average cell size for a phytoplankton SDS of -3.5 to -4 is ~1.2 μm, giving an average distance between cells of ~35 body lengths. To convert this value to distances between tree roots, we assumed a 12 m high deciduous tree with a crown diameter of 9 m. For such trees, the rooting system typically has a diameter 2 to 3 times that of the crown diameter (https://deepgreenpermaculture.com/2019/07/22/how-far-do-large-tree-roots-extend/), giving for our calculation a root diameter of ~23 m. Applying the relative body length spacing between individual phytoplankton calculated above yields an equivalent spacing of order ~1 km between the roots of neighboring trees of equal size. A significantly greater distance between trees results if we apply a body length spacing calculated using more typical phytoplankton concentrations of 10^{11} to 10^{12} cells m^{-3}.

Ecosystem Modeling

The model equations used in the current study are based on the pioneering work of Riley et al. [1949], Margalef [1968], and Evans and Parslow [1985] and were used by the current authors in previous analyses of phytoplankton blooms [Behrenfeld and Boss 2014, 2018]. If for these model equations we initially assume that herbivores equally graze phytoplankton of all size classes, the equation for phytoplankton biomass of size class, *i*, will be (equation 1 in main text):

$$\frac{dP_i}{dt} = \mu P_i - c_{1,i} P_i H \quad (1)$$

and the equation for herbivore biomass will be (equation 2 in main text):

$$\frac{dH}{dt} = c_{1,i} c_2 H \sum P_i - c_3 H^2 \ . \quad (2)$$

The steady-state solutions for (1) and (2) are $(P_i,\ H) = \left(\frac{c_3 \mu}{c_{1,i}{}^2 c_2}, \frac{\mu}{c_{1,i}}\right)$, which implies that the biomass of phytoplankton and grazers are proportional to phytoplankton growth rate (μ).

If we now assume that the parameters in (1) and (2) (i.e., μ, c_1, c_2, and c_3) are size-independent, then the biomass of all phytoplankton size classes is the same such that $P_i = \frac{1}{M} \sum P_i = \frac{c_3 \mu}{c_1{}^2 c_2}$, where *M* is the number of phytoplankton size classes. Since this solution is for biomass, the abundance of cells in each size class ($N_{p,i}$) changes as a function of biomass divided by cell volume ($N_{p,i} \sim \frac{const}{D_i^3}$), which gives a SDS of -3.

To account for the observed fundamental SDS of -4, it is necessary to consider an additional trophic phenomenon, specifically that different herbivores (H_k) have different prey ranges for phytoplankton (P_i). This consideration modifies equations (1) and (2) as:

$$\frac{dP_i}{dt} = \mu P_i - \sum_i c_{i,k} H_k P_i \quad (3)$$

$$\frac{dH_k}{dt} = c_2 \sum_k c_{i,k} P_i H_k - d_k {H_k}^2 , \quad (4)$$

where the $c_{i,k}$ parameters are constants greater than zero if H_k consumes phytoplankton of size P_i and are otherwise zero if the phytoplankton size is outside the feeding range of H_k. As discussed in the main text, a simple expectation is that the prey size range is proportional to the mean prey size within that range (e.g. if the diameter of the mean prey size is D_i, then the herbivore will consume phytoplankton over a size range of $\alpha D_i \geq D_i \geq \frac{D_i}{\alpha}$, where α is the proportionality factor ($\alpha > 1$)). If we denote H_i as all herbivores that consume phytoplankton of bin size, i, and these bins scale with the prey size as described above, we can rewrite equations (3) and (4) as:

$$\frac{dP_i}{dt} = \mu P_i - c_1 P_i H_i \quad (5)$$

$$\frac{dH_i}{dt} = c_1 c_2 H_i P_i - c_3 {H_i}^2 , \quad (6)$$

giving steady-state solutions for (5) and (6) that are again $(P_i,\ H_i) = \left(\frac{c_3 \mu}{{c_1}^2 c_2}, \frac{\mu}{c_1}\right)$. Solving these steady-state solutions (again with size-independent values for μ, c_1, c_2, and c_3) for each size bin, i, where a fixed proportionality is assumed between mean prey diameter and prey diameter range, and then dividing the phytoplankton continuum into sequential prey bins of proportionally increasing size, yields an equal biomass within each increasing size bin [a result consistent with observations of Sheldon et al. 1972 and citations therein]. Such a distribution gives a fundamental SDS of -4.

The stability of this fundamental SDS can be evaluated by relaxing the assumption of size-independent values for μ, c_2, and c_3. The outcome of this evaluation is shown by the blue shading in figure 1b of the main manuscript. Here, we first allowed μ to vary logarithmically

with phytoplankton size, such that the value of μ for a 100 µm phytoplankton was 60% lower than that of 1 µm cell. This modification yielded a SDS of -4.1. We then allowed c_3 to logarithmically decrease with size by 60% between 1 and 100 µm, yielding a further tipping of the SDS to -4.4. Finally, allowing ingestion efficiency (c_2) to increase a factor of two between 1 and 100 µm cells pushed the SDS to -4.6. While the actual size-dependency of μ, c_2, and c_3 in nature is poorly constrained, what this evaluation demonstrates is that the fundamental SDS is relatively resilient to variations in how these terms are parameterized and also that variations in this parameterization can accommodate much of the observed, albeit constrained, SDS variability in temporally-stable aquatic environments (figure 1b of the main manuscript).

Supplemental References

Behrenfeld, M.J., Boss, E.S. Resurrecting the ecological underpinnings of ocean plankton blooms. *Ann. Rev. Mar. Sci.* 2014; **6,** 167–94.

Behrenfeld, M.J., Boss, E.S. Student's tutorial on bloom hypotheses in the context of phytoplankton annual cycles. *Global Change Biol*. 2018; **24,** 55-77.

Evans, G. T., Parslow, J. S. A model of annual plankton cycles. *Biological Oceanogr.* 1985; **3,** 327–347.

Margalef, R. *Perspectives in ecological theory*. Univ. Chicago Press, Chicago, Ill. 1968; 111 pp.

Riley, G.A., Stommel, H.M., Bumpus, D.F. Quantitative ecology of the plankton of the western North Atlantic. *Bull. Bingham Oceanogr*. 1949; Collect. 12, Yale Univ., New Haven, CT.

Sheldon, R.W., Prakash, A., Sutcliffe Jr, W. The size distribution of particles in the Ocean 1. *Limnol. Oceanogr.* 1972; **17,** 327-340.
